# Meeting Cancer Detection Benchmarks in MRI/Ultrasound Fusion Biopsy for Prostate Cancer: Insights from a Retrospective Analysis of Experienced Urologists

**DOI:** 10.3390/cancers17020277

**Published:** 2025-01-16

**Authors:** Fabian Utzat, Stefanie Herrmann, Matthias May, Johannes Moersler, Ingmar Wolff, Johann Lermer, Mate Gregor, Katharina Fodor, Verena Groß, Anton Kravchuk, Thomas Elgeti, Stephan Degener, Christian Gilfrich

**Affiliations:** 1 Department of Urology, University of Witten/Herdecke, 42283 Wuppertal, Germany; fabian.utzat@uni-wh.de (F.U.); stephan.degener@uni-wh.de (S.D.); 2 Department of Urology, St. Elisabeth Hospital Straubing, 94315 Straubing, Germany; matthias.may@klinikum-straubing.de (M.M.); johann.lermer@gmail.com (J.L.); katharina.fodor@klinikum-straubing.de (K.F.); verena.gross@klinikum-straubing.de (V.G.); anton.kravchuk@klinikum-straubing.de (A.K.); christian.gilfrich@klinikum-straubing.de (C.G.); 3 Department of Radiology, St. Elisabeth Hospital Straubing, 94315 Straubing, Germany; johannes.moersler@klinikum-straubing.de; 4 Department of Urology, University Medicine Greifswald, 17475 Greifswald, Germany; ingmar.wolff@med.uni-greifswald.de; 5 Institute of Pathology, Hospital Deggendorf, 94469 Deggendorf, Germany; dr.m.gregor@pathologie-deggendorf.de; 6 Department of Radiology, Charité-Universitätsmedizin Berlin, Corporate Member of Freie Universität Berlin and Humboldt-Universität zu Berlin, 12203 Berlin, Germany; thomas.elgeti@charite.de

**Keywords:** learning curve, image-guided biopsy, targeted biopsy, systematic biopsy, fusion biopsy, urological surgical procedures, multiparametric MRI, mpMRI, PI-RADS, patient safety

## Abstract

Background: The relationship between case volume and clinical outcomes is well established for most urological procedures but remains underexplored in prostate ultrasound/MRI fusion biopsy (UMFB). UMFB aims to detect clinically significant prostate cancer (csPCa) by adhering to cancer detection benchmarks for PI-RADS lesions identified via multiparametric MRI (mpMRI). These benchmarks, defined by Ahmed et al., include cumulative cancer detection rate (C-CDR) targets of >80% for PI-RADS 5, >50% for PI-RADS 4, and <20% for PI-RADS 1–3. Methods: This retrospective, single-center study analyzed the case volumes required for two experienced urologists (U1 and U2, each with >15 years of practice) to consistently achieve the Ahmed-defined C-CDR benchmarks for csPCa (ISUP grade ≥ 2) using UMFB. Both transrectal and transperineal approaches were included to enable comprehensive learning curve analysis. Data from 2017 to 2023 were reviewed, encompassing 157 UMFBs performed by U1 and 242 by U2, with a transrectal-to-perineal ratio of 7:3. Results: Both urologists achieved Ahmed-defined C-CDR targets from the outset. Over a median follow-up of 30 months, patients with initial PI-RADS 4 or 5 ratings and negative primary biopsies remained prostate cancer-free in 77% of cases for U1 and 91.2% for U2 (*p* = 0.152). Conclusions: This study demonstrates that experienced urologists can achieve high diagnostic accuracy and maintain patient safety immediately upon implementing UMFB, meeting established benchmarks without requiring additional procedural learning.

## Simple Summary

This study examines how quickly experienced urologists learn to perform a specialized prostate biopsy method that combines MRI and ultrasound to detect prostate cancer. This technique, called ultrasound/MRI fusion biopsy (UMFB), enhances the detection of aggressive cancers while helping to avoid unnecessary treatment for less harmful cases. We observed two urologists, each with over 15 years of experience, and found that both doctors met high standards for cancer detection from the outset, showing no need for an extended learning period. This study also shows that combining two biopsy methods—targeted and systematic—was more effective in identifying significant cancers. Regular patient follow-ups further verified the accuracy of the biopsy results. Overall, this study highlights that with proper training and teamwork, UMFB can be safely and effectively implemented from the beginning, improving diagnostic outcomes for prostate cancer patients.

## 1. Introduction

Prostate cancer is the second most commonly diagnosed cancer among men globally, accounting for one in three cancer-related deaths in Europe. Despite its prevalence, clinically insignificant prostate cancer (cisPCa) remains a significant risk factor for overdiagnosis and overtreatment [1]. Detecting clinically significant PCa (csPCa) remains a central challenge in urology. The integration of multiparametric MRI (mpMRI) with ultrasound/MRI fusion biopsy (UMFB) significantly improves diagnostic accuracy by reducing the detection of cisPCa [2,3,4].

Despite evident advancements in imaging analysis and fusion technology, diagnostic outcomes are still influenced by the expertise of the radiologist interpreting the mpMRI and the urologist performing the biopsy. Although multiple studies have explored the relationship between case volume and cancer detection rates (CDRs) in UMFB, the findings remain remarkably heterogeneous. For example, Gereta et al. found no indication of an operator-dependent learning curve, while other studies observed a strong association between case volume and CDR [5,6,7]. Notably, studies suggesting a learning curve effect identified varying case volumes required to stabilize CDR levels, typically in the range of 50 to 100 UMFBs [6,7]. Two key factors complicate comparisons across studies on learning curve effects: (i) the target CDR levels varied significantly, with some studies including all PCa and others focusing solely on csPCa, and (ii) methodological differences in assessing learning curve effects were substantial [6,7,8]. In a recent study involving 107 patients undergoing UMFB, Xu et al. concluded that adequate proficiency could be attained after approximately 50 UMFBs, establishing target CDRs for csPCa at > 80% for PI-RADS 5, > 50% for PI-RADS 4, and < 20% for PI-RADS ≤ 3 [8]. This CDR framework, based on Ahmed et al.’s work, was first used by Xu et al. to analyze learning curve effects [4,8].

This study aims to evaluate the existence of a learning curve for UMFB in two seasoned urologists who were newly introduced to UMFB, within a retrospective single-center cohort, based on the predefined detection criteria by Ahmed et al. [4]. Additionally, we provide follow-up data for patients with negative biopsy results and analyze Gleason upgrading rates in specimens from subsequent robot-assisted radical prostatectomy (RARP).

## 2. Materials and Methods

### 2.1. Study Population and Design

This retrospective single-center study included all patients who underwent UMFB (via either transrectal or transperineal approach) at St. Elisabeth Hospital Straubing from January 2017 to September 2023 (68 months). Patients were included if they had clinical suspicion of PCa, defined by a prostate-specific antigen (PSA) level ≥ 3 ng/mL and/or an abnormal digital rectal examination (DRE), and subsequently underwent mpMRI with at least one PI-RADS ≥3 lesion. This cohort included patients with prior negative biopsy results but persistent PCa suspicion. Exclusion criteria were a patient-withdrawn informed consent, incomplete or missing mpMRI results, and a prior PCa diagnosis. For patients with PI-RADS 4 or 5 lesions and initially negative biopsy results, follow-up data (PSA levels, subsequent mpMRI findings, or additional biopsy results) were collected through their outpatient urologists or available in-house records. Patients with confirmed PCa who underwent subsequent RARP were screened for Gleason score upgrading upon final histopathology of the prostate.

The data analyzed were collected within the scope of patients’ standard clinical care agreements, with obtained ethics approval from the Ethics Committee of the Bavarian State Chamber of Physicians (Approval No. 2023–1139, 26 September 2023) and additionally confirmed by the Ethics Committee of the University of Witten/Herdecke (Approval No. S-250/2023, 12 October 2023). Approval by both committees confirmed that individual patient consent was not required, as exclusively medical data collected through routine clinical care were used.

### 2.2. MRI and Biopsy Protocol

Multiparametric MRI was performed using 1.5 or 3T systems with multichannel phased-array surface coils, following the guidelines of the European Society of Urogenital Radiology (ESUR) [9]. The imaging was conducted either at certified external radiology centers or in-house at a specialized facility. DICOM data from external mpMRIs were imported into the in-house Picture Archiving and Communication System (PACS). Both external and in-house mpMRIs included T2-weighted, diffusion-weighted, and dynamic contrast-enhanced imaging, which were all interpreted by a certified mpMRI radiologist (J.M., with eight years of prostate mpMRI experience at this study’s outset) and classified according to PI-RADS v2 guidelines [10]. Lesions graded as PI-RADS 3–5 were identified prior to ultrasound fusion, with our radiologist marking the configuration of the regions of interest (ROI) for each mpMRI (internal and external) to provide standardization.

The choice between transrectal and transperineal approaches was based on patient preference and individual risk factors, such as lesion location. Transrectal ultrasound fusion-guided biopsy (UMFB) was performed under local anesthesia (22 G needle, 20 mL of 1% lidocaine) or general anesthesia, while the transperineal UMFB was conducted solely under general anesthesia. Peri-interventional antibiotic prophylaxis (PAP) adhered to current guidelines or employed a targeted approach based on pathogen identification from urine or rectal cotton swab culture.

UMFB was performed using a mobile ultrasound diagnostic device (Medcom GmbH, Darmstadt, Germany), the 3D Guidance trakSTAR system version 3.3 (NDI Europe, Radolfzell, Germany), a Microstepper MST 150 (GFM GmbH, Gross-Gerau, Germany), and an E11C3b transrectal ultrasound probe (BK Medical Medizinische Systeme GmbH, Quickborn, Germany). mpMRI-fused ultrasound facilitated adjustments in transverse and sagittal imaging. A biopsy gun (18 G, 22 mm specimen length, Bard Magnum, Bard Medical, Covington, KY, USA) was used to obtain 2–4 targeted biopsy (TB) cores per lesion, with systematic biopsies (SBs) taken from 12 predefined prostate areas in accordance with the Ginsburg protocol [11]. UMFB results were reported in alignment with the START (Standards of Reporting for MRI-targeted Biopsy Studies) checklist [12].

### 2.3. Histopathological Analysis

All specimens were fixed in labeled formalin containers and analyzed by the in-house pathology department. Histopathological evaluations were supervised by a pathologist with 10 years of experience in prostate biopsy assessment (M.G.). PCa was classified according to the 2014 “International Society of Urological Pathology (ISUP) Consensus Conference on Gleason Grading of Prostatic Carcinoma”, dividing cases into five ISUP grades [13]. csPCa was defined as ISUP grade ≥ 2 (Gleason score ≥ 3 + 4), while cisPCa was classified as ISUP grade 1 (Gleason score 3 + 3). For patients undergoing subsequent RARP, whole-mount sectioning was used for histopathological analysis, with a re-evaluation of ISUP grading. Gleason upgrading was defined as an increase in ISUP grade in the prostatectomy specimen compared to the biopsy findings from both TB and SB.

### 2.4. Data Collection and Study Objectives

Data collected included patient demographics (age at biopsy, prostate volume measured by mpMRI, and PSA levels before biopsy) and procedural details (biopsy approach: transperineal or transrectal). Data collection adhered to the START checklist, and ISUP grades were recorded for TB, SB, and RARP specimens when available. During this study, UMFB was performed by 17 surgeons. Based on prior research, only surgeons who completed at least 50 UMFBs were included in the analysis [7,8]. Two urologists (U1 and U2), each with over 15 years of ultrasound-guided prostate biopsy experience, met this criterion.

The primary study objective was to analyze the cumulative CDR (C-CDR) within the context of UMFB and to determine whether an individual learning curve was necessary for each urologist to achieve a predefined stable level in the detection of csPCa across various PI-RADS lesions. These benchmarks were defined as CDRs of 80% for PI-RADS 5, 50% for PI-RADS 4, and < 20% for PI-RADS 3, following Ahmed et al. [4]. Additionally, we provided a comparison of csCDR and overall CDRs divided for PI-RADS, concerning transrectal vs. transperineal biopsy approach and internally vs. externally performed mpMRI.

To further assess diagnostic reliability, follow-up data were analyzed for patients with negative biopsies but PI-RADS 4 or 5 lesions on mpMRI. A negative follow-up was defined as repeated negative biopsy, repeated negative mpMRI, or consistently decreasing PSA levels. Additionally, Gleason upgrading was assessed by comparing biopsy findings to the final histopathology from RARP specimens.

The secondary study objective was to evaluate the added diagnostic value of SB in detecting csPCa compared to TB alone or the combination of TB and SB.

### 2.5. Statistical Analysis

Continuous variables are presented as medians with interquartile ranges (IQRs), while categorical variables are expressed as absolute counts and percentages. Group comparisons for continuous variables were conducted using the Mann–Whitney U test, while categorical variables were analyzed using either the chi-square test or Fisher’s exact test. To assess urologist performance, biopsies were grouped into increments of 25 for the initial step and increments of 50 cases thereafter, with individual detection rates for csPCa compared using Fisher’s exact test. To evaluate whether observed csPCa-to-PI-RADS lesion ratios for each urologist significantly deviated from Ahmed et al.’s predefined proportions [4], a series of two-sided binomial tests were conducted. Each ratio at predefined cumulative case count thresholds was assessed against the hypothesized proportions of 0.2 (20%) for PI-RADS 3, 0.5 (50%) for PI-RADS 4, and 0.8 (80%) for PI-RADS 5, as established by Ahmed and relevant to each threshold.

For each binomial test, the observed number of csPCa cases was compared to the total sample size for each PI-RADS lesion. A two-sided *p*-value was derived to determine whether observed proportions differed significantly from expected values, with statistical significance set at *p* < 0.05. Statistical analyses were performed using SPSS 29.0 (IBM Corp., Armonk, NY, USA).

## 3. Results

### 3.1. Descriptive Results

During the study period, 994 biopsies (transrectal or transperineal) were performed at our center, averaging 15 procedures per month. Of these, 581 patients met the inclusion and exclusion criteria, forming the study cohort.

Patient demographics showed a median age of 70 years (IQR: 64–75), a median prostate volume of 49 cm³ (IQR: 36–68), and a median PSA level of 7.3 ng/mL (IQR: 5.3–10.6). The majority of biopsies (69.7%, *n* = 405) were conducted transrectally, with the remaining 30.3% performed using the transperineal approach.

Among all mpMRIs performed, 53.5% were conducted in-house. In total, 731 lesions classified as indeterminate or suspicious for prostate cancer were identified: of the primary lesions, 6.4% (*n* = 37) were rated as PI-RADS 3, 64.4% (*n* = 374) as PI-RADS 4, and 29.3% (*n* = 170) as PI-RADS 5. Additionally, 18.4% (*n* = 107) of mpMRIs identified two or more lesions categorized as PI-RADS 4 or 5.

**Figure 1:**
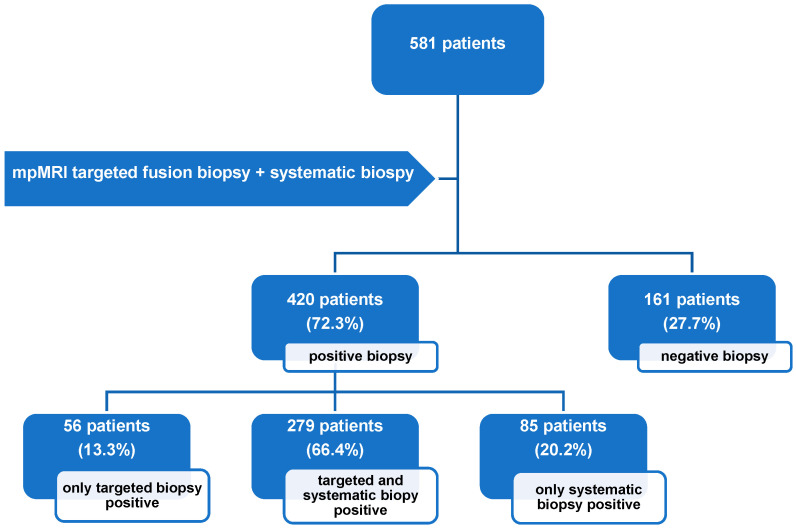
Flowchart illustrating biopsy outcomes for patients who underwent combined UMFB during the observation period. Positive biopsy results indicate detection of either clinically significant prostate cancer or clinically insignificant prostate cancer. mpMRI: multiparametric magnetic resonance imaging.

The overall cancer detection rate (CDR) for PCa was 72.3% (*n* = 420). Among these cases, 20.2% (85/420) were detected exclusively by SB, and 13.3% (56/420) were identified solely by TB (Figure 1). For csPCa, a CDR of 54.7% (318/581) was found: 14.5% (46/318) of csPCa cases were detected by SB alone, 12.3% (39/318) by TB alone, and 73.3% (233/318) were detected in both approaches.

### 3.2. Learning Curve Analysis

During the study period, 581 UMFB procedures were performed by 17 urologists. Of these, 15 urologists performed 182 biopsies, averaging 12 biopsies per urologist, while the remaining 399 UMFBs (68.7%) were conducted by two urologists, U1 and U2, who were selected for individual learning curve analysis. U1 performed 157 biopsies (27.0% of the total), and U2 performed 242 biopsies (41.7%). Table 1 provides a comparative overview of patient characteristics and histopathological results for both urologists. Comparing the patients of both urologists (U1 and U2) according to the biopsy approach (transrectal or transperineal), no significant difference in csCDR and overall CDR was found for PI-RADS 3–5, as displayed in Table 2. Additionally, comparing internal performed mpMRIs with external ones for the 399 patients of both urologists, again, no significant differences in csCDR and overall CDR divided for PI-RADS 3–5 were found (Table 3).

For learning curve analysis, biopsy cases were divided into steps of 25 or 50, and the C-CDR of csPCa was compared for both urologists by PI-RADS categories. No significant differences were found between U1 and U2 (Table 4).

**Figure 2:**
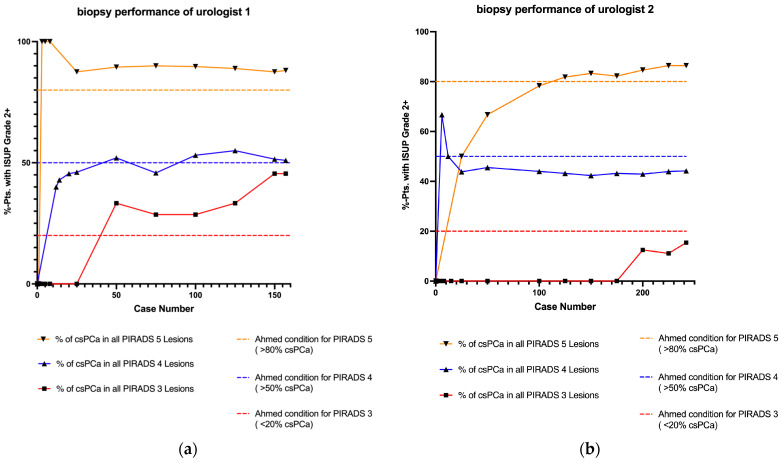
Learning curves for UMFB displayed according to Ahmed’s criteria [4] and stratified by PI-RADS scores. No significant deviations from Ahmed’s criteria were observed for either urologist. (**a**) Learning curve for Urologist 1. (**b**) Learning curve for Urologist 2. csPCa: clinically significant prostate cancer; ISUP: International Society of Urological Pathology; PI-RADS: Prostate Imaging Reporting and Data System; pts: patients; UMFB: ultrasound/MRI fusion biopsy of the prostate.

For U1, the Ahmed criteria [4] were unmet at case volume 25 for PI-RADS 4 due to underperformance and at case volumes 50, 100, and 150 for PI-RADS 3 due to overperformance. At the final case volume, U1 demonstrated an overall over-detection rate in PI-RADS 3 lesions, with a csPCa rate of 45.5%. For U2, the criteria were not met for PI-RADS 4 at case volumes 25, 50, 100, and 150, while underperformance was observed at case volumes 25, 50, and 100 for PI-RADS 5. At the final case volume, U2 showed an overall underperformance for PI-RADS 4, with a csPCa detection rate of 44.2% (Table 4). To verify the statistical significance of these deviations, a two-sided binomial test was conducted, as shown in Table 5. The results indicated no significant deviation from the Ahmed criteria (the individual *p*-values ranged from 0.050 to 0.999). Overall, neither urologist required a stabilization phase indicative of a learning curve to achieve the expected performance levels. Figure 2 illustrates the described performance outcomes.

### 3.3. Follow Up Results

Of the patients presenting with an initial PI-RADS 4 or 5 lesion on mpMRI, 92 had negative biopsy results from UMFB. Follow-up data were available for 83 of these patients, with a median follow-up of 30 months (IQR: 15–51), while follow-up data were unavailable for 9.8% (9/92) of the cases.

Among the 157 UMFB procedures performed by Urologist 1 (U1), 36 cases (23%) showed no evidence of PCa, with 30 of them involving an initial PI-RADS 4 or 5 lesion. Of these 30 patients with an initially negative biopsy result, 4 patients (13%) were lost to follow-up. For the remaining 26 patients, the median follow-up period was 45 months, during which 20 (77%) showed no evidence of PCa (Table 1).

For Urologist 2 (U2), 28.5% (69/242) of biopsies were negative for PCa. Of these 69 patients with initially negative results, 62 had an initial PI-RADS 4 or 5 lesion, with follow-up data available for 57 patients (median follow-up of 23 months), reflecting a follow-up loss rate of 8%. Among these 57 patients, 52 (91.2%) showed no evidence of PCa during follow-up (Table 1).

There was no significant difference between the two urologists in detecting PCa among patients within the follow-up associated with an initial PI-RADS 4–5 lesion and an initial negative histology (23% vs. 8.8% for U1 and U2, respectively; *p* = 0.152). Additionally, Gleason upgrading rates were assessed for patients who underwent subsequent RARP following diagnosis of PCa. Data were available for 140 cases, with comparable upgrading rates between both surgeons: 16.7% (10/60) for U1 and 18.8% (15/80) for U2 (*p* = 0.826).

## 4. Discussion

This study aimed to assess whether urologists, proficient in biopsy procedures but newly introduced to UMFB, faced a learning curve in order to achieve satisfactory csPCa detection rates. To this end, we conducted a retrospective single-center study with a robust sample size (*n* = 581) that included follow-up data. Our findings demonstrate that two experienced urologists, who were thoroughly trained and proctored in UMFB and operated within a fixed setup alongside seasoned radiologists and pathologists, achieved satisfactory csPCa detection rates from the outset, without requiring a learning period. These results emphasize the potential of UMFB to achieve high diagnostic standards from the start when performed by skilled urologists, suggesting that adoption of UMFB could be streamlined in other centers through structured training and close interdisciplinary collaboration, thereby enabling immediate enhancements in prostate cancer diagnostic outcomes.

Among studies examining UMFB learning curves, the use of clearly defined percentage thresholds for csPCa detection, categorized by specific PI-RADS lesions, is an uncommon feature. Inspired by Xu et al., our study adhered to the British working group’s quality benchmarks as outlined by Hashim Ahmed et al., which set meaningful thresholds for diagnostic outcomes [4,8]. Ahmed’s CDR benchmarks comprise two essential components [4]: achieving csCDRs >80% and >50% for PI-RADS 5 and 4 lesions, respectively, underscoring both biopsy accuracy and radiological proficiency in interpreting multiparametric MRI (mpMRI). Additionally, restricting csPCa detection in PI-RADS 1–3 lesions to below 20% highlights the necessity for precise mpMRI interpretation. Importantly, PI-RADS 1–3 outcomes are less pertinent when evaluating the urologist’s learning curve.

For Urologist 1, the learning curve demonstrated a stable trajectory for PI-RADS 4 and 5 lesions, with no significant learning effect. Minor deviations in the PI-RADS 4 data relative to the 50% benchmark established by Ahmed did not reach statistical significance. In PI-RADS 3, Urologist 1 displayed an overperformance in csPCa detection without statistical significance. Assuming Ahmed’s criteria are valid, the elevated detection rate in PI-RADS 3 could suggest radiological misclassification. Conversely, if the radiological assessment was accurate, Ahmed’s classification criteria might warrant revision, as PI-RADS 3 findings could merit closer investigation. For Urologist 2, a linear but suboptimal trajectory was observed in PI-RADS 4; however, this deviation from the threshold was not statistically significant. For PI-RADS 5, the learning curve crossed the 80% threshold after approximately 100 cases, suggesting a potential learning effect, though no significant deviation from Ahmed’s benchmarks was noted. Both urologists met the predefined criteria from the outset, although our analysis did not include PI-RADS 1 and 2, as in Ahmed’s 20% threshold, which may account for the trend towards overperformance. Defining “overperformance” in detecting csPCa, however, may lack clinical relevance.

The current literature on UMFB learning curves is notably heterogeneous. To contextualize our findings, we synthesized outcomes from studies published between 2017 and 2024 (Table 6), which reveal variability in the presence of a learning curve for csPCa detection using MRI/US fusion-guided biopsy. Ten studies identified a significant learning curve effect, with plateau points ranging from 25 to 500 cases, where csPCa detection rates improved with experience [7,8,14,15,16,17,18,19,20,21]. Conversely, another ten studies found no learning curve effect, indicating that csPCa detection proficiency was either immediate or consistent across experience levels [5,22,23,24,25,26,27,28,29,30]. Notably, aside from Lenfant et al. and Xu et al. [7,8], few studies defined quality criteria for CDR. Interpreting a learning curve merely as an increase in CDR may not indicate substantial improvement. Furthermore, most studies did not report CDRs by PI-RADS category, complicating direct comparison with our findings. Comparable in methodological granularity to our study, Himmelsbach et al. and Hsieh et al. reported cancer detection rates (CDRs) of clinically significant prostate cancer (csPCa) by PI-RADS category, with Himmelsbach’s study yielding 21.2% for PI-RADS 3, 44.5% for PI-RADS 4, and 80.1% for PI-RADS 5, while Hsieh’s reported rates of 28.9%, 51.1%, and 85.7%, respectively [15,23]. These data align with Ahmed’s benchmarks in PI-RADS 5 and in PI-RADS 4 for Hsieh et al., although elevated PI-RADS 3 rates may imply radiological underestimation. Meng et al. [16] similarly reported csCDRs by PI-RADS category, meeting Ahmed’s threshold only in PI-RADS 3 (6% for PI-RADS 3, 46% for PI-RADS 4, and 66% for PI-RADS 5). This variability underscores the complexities inherent in learning curve research for UMFB, where study design, biopsy software, devices, and endpoints limit comparability. Detection rates for csPCa range from 23.2% to 100% across biopsy systems [31], substantially affecting outcomes.

Surgeon experience is a crucial factor. In our study, Urologists 1 and 2 had over 15 years of experience in TRUS-guided biopsies. Calleris et al. found that experienced surgeons can achieve competency in UMFB from the outset, consistent with our findings [24]. Multiple studies support a positive correlation between experience and CDR [16,17,32]. Lenfant et al. also demonstrated that urologists with varying expertise levels exhibit different learning curves, further emphasizing experience’s impact on UMFB proficiency [7]. The quality of training and proctoring is likewise critical: Urologist 1 received training from the UMFB device manufacturer, while Urologist 2 was mentored by Urologist 1. Studies by Cata et al. and Berg et al. confirm that mentoring, MRI training, and guidance from skilled surgeons enable novices to quickly achieve UMFB competence [18,22]. Finally, interdisciplinary collaboration is essential to optimal outcomes. Collaboration between urologists, radiologists, and pathologists enhances csPCa detection while minimizing cisPCa overdiagnosis. Urkmez et al. advocate a closed-loop learning system involving expert input to improve outcomes [19]. At our center, experienced radiologists and pathologists significantly contributed to biopsy accuracy, with our radiologist, certified in mpMRI, having reviewed over 100 cases prior to the start of the study, surpassing the expertise level Moore et al. recommend (50–100 cases) [12]. Our high biopsy quality reflects this interdisciplinary approach and rigorous proctoring.

Assessing biopsy quality is also imperative. For example, Xu et al. [8] relied solely on PI-RADS, which may be problematic given mpMRI’s high sensitivity (87%) but low specificity (41%), yielding potential false positives [4]. This limitation underscores the need for additional validation tools, as false-positive PI-RADS 4/5 findings may incorrectly suggest inadequate biopsy performance. Our follow-up data align with the literature, with a 70.3% negative follow-up rate in PI-RADS 4/5 cases reported by Kornienko et al. [33]. Similarly, our Gleason upgrading rates (16.7% for U1, 18.8% for U2) are consistent with Arsov et al. (11.5–28.8%) and Lee et al. (7–22%) [34,35]. The low missed PCa rate and stable Gleason grading in our study suggest reliable biopsy data.

Our overall CDR of 72.3% for all patients is quite high compared to two randomized controlled trials: the PRECISION study [2] reported a csCDR of 38% and a cisCDR of 9%, while the FUTURE trial [36] described overall CDRs between 44% and 55%. However, similar results are reported in more recent studies: Gereta et al. [5] found overall CDRs between 61% and 71% and Alargkof et al.’s [28] chief resident detected PCa in 71.4%, aligning with our excellent detection rates.

With respect to our secondary study objectives, SB identified 14.5% of csPCa cases that would have been missed by TB alone, aligning with Filson et al.’s 16% csPCa detection rate in MRI-negative cases [37]. Although SB’s diagnostic value is debated, a Cochrane review by Drost et al. found that MRI-guided biopsy alone enhances csPCa detection while reducing cisPCa overdiagnosis [38]. However, Rouviere et al. and Ahdoot et al. showed that combining SB and TB enhances csPCa detection and reduces postoperative upgrading after RARP [39,40]. The German S3 guidelines recommend this combination for biopsy-naive patients (Grade B recommendation) [41].

Comparing transperineal and transrectal biopsy approach, our data showed no significant difference for csPCa and overall CDR, in line with Kaneko et al. [42]. In contrast, Oderda et al. [43] demonstrated a significantly higher csCDR for transperineally UMFB, but again found no difference for overall CDR.

Our study has limitations that should be considered when interpreting the results: This study is a non-randomized, non-controlled retrospective analysis conducted at a single certified prostate cancer center. This design inherently limits the generalizability of our findings and may introduce selection bias related to patient demographics and clinical practices. While our results align with those of other single-center studies, caution should be exercised when extrapolating these findings to broader clinical settings.

In addition, we excluded urologists with low UMFB case numbers (<50 cases) and analyzed the urologists sequentially. Our study solely focused on evaluating potential learning curves among the urologists at our institution who had performed at least 50 cases of UMFB from the start of their individual experience. Additionally, the inclusion of only experienced urologists might have distorted our results, as there might be variable diagnostic performance across different experience levels, resulting in limited generalizability for less experienced physicians, as they might have a flatter learning curve.

In this context, we would like to highlight that our learning curve analysis focused exclusively on oncological outcomes, without incorporating other procedural quality aspects such as procedure time, complication rates, or infection rates. This approach was chosen to maintain a clear and specific focus in our study. Furthermore, a recent study by Mian et al. [44] demonstrated that there do not appear to be any significant differences in complication rates between the transperineal and transrectal biopsy approaches, supporting the rationale for not differentiating these approaches in terms of complication rates in our analysis.

Next, external imaging may have caused variability concerning imaging quality, although there was a local review of all mpMRIs/ROIS. But in order to assess individual learning curves, it is also crucial to include external mpMRIs, as this reflects real-life conditions.

Finally, follow-up data were missing for 9.8% of PI-RADS 4/5 cases with negative biopsies, and U1 had a longer median follow-up period.

## 5. Conclusions

This study demonstrates that experienced urologists newly trained in UMFB can achieve satisfactory detection rates for clinically significant prostate cancer (csPCa) from the outset, given a structured training program and close interdisciplinary collaboration with experienced radiologists and pathologists. Our findings suggest that implementing a similar approach in other clinical centers may enable a rapid, reliable adoption of UMFB, thus enhancing diagnostic accuracy in prostate cancer. While our study highlights the feasibility of achieving high diagnostic standards without an extended learning curve, further research across diverse settings is needed to validate these findings and to optimize UMFB training frameworks. Additionally, consideration of standardized quality benchmarks, such as those by Ahmed et al. [4], could aid in establishing consistent practices for csPCa detection. These results underscore the value of a collaborative, structured approach in modern prostate cancer diagnostics, with UMFB offering a viable pathway towards improved clinical outcomes from the very start.

## Figures and Tables

**Table 1:** Comparison of clinical and histopathological characteristics of patients assessed by two urologists (U1 and U2) in relation to their learning curves with UMFB (*n* = 399).

Parameter	U1 (*n* = 157)	U2 (*n* = 242)	*p*
Median age, years (IQR)	71 (65–76)	68 (63–73.3)	**<0.001**
Median PSA, ng/mL (IQR)	7.0 (5.3–10.8)	7.8 (5.6–11.1)	0.134
Median prostate volume, cm^3^ (IQR)	45.3 (35.0–64.4)	51.9 (35.0–73.8)	0.225
Proportion of PCa, *n* (%)	121 (77.1)	173 (71.5)	0.216
Proportion of csPCa, *n* (%)	95 (60.5)	131 (54.1)	0.209
Proportion of PCa solely detected by SB, *n* (%)	22 (14.0)	38 (15.7)	0.726
Proportion of csPCa solely detected by SB, *n* (%)	15 (9.6)	17 (7.0)	0.550
Median FU of pts. with negative biopsy and PI-RADS 4/5, months (IQR)	45 (18–51)	23 (15–31)	**<0.001**
Number of pts. with PI-RADS 4/5 and negative biopsy, *n* (%)	30 (19.1)	62 (25.6)	0.425

Abbreviations: csPCa: clinically significant prostate cancer; FU: follow-up; IQR: interquartile range; PI-RADS: Prostate Imaging Reporting and Data System; PSA: prostate-specific antigen; pts: patients; SB: systematic biopsy; UMFB: ultrasound/MRI fusion biopsy of the prostate. Significant differences below an alpha error threshold of 5% were highlighted in bold.

**Table 2:** Comparison of PCa and csPCa CDR differentiated by transrectal and transperineal approach for the patients biopsied by Urologist 1 and Urologist 2 (*n* = 399).

	Transrectal Biopsies *n*/*n* (%)		Transperineal Biopsies *n*/*n* (%)		*p*
PCa in PI-RADS 3	8	(50%)	3	(37.5%)	0.679
csPCa in PI-RADS 3	6	(37.5%)	1	(12.5%)	0.352
PCa in PI-RADS 4	131	(67.2%)	52	(72.2%)	0.461
csPCa in PI-RADS 4	90	(46.2%)	35	(48.6%)	0.783
PCa in PI-RADS 5	73	(90.1%)	27	(100%)	0.197
csPCa in PI-RADS 5	68	(84%)	26	(96.3%)	0.182

Abbreviation: CDR: cancer detection rate; csPCa: clinically significant prostate cancer; PCa: prostate cancer; PI-RADS: Prostate Imaging Reporting and Data System; *p*: *p*-values representing the statistical significance of differences between transrectal and transperineal approach.

**Table 3:** Comparison of PCa and csPCa CDR differentiated by modus of MRI (intern/extern) for the patients biopsied by Urologist 1 and Urologist 2 (*n* = 399).

	Biopsies with Intern MRI *n*/*n* (%)		Biopsies with Extern MRI *n*/*n* (%)		*p*
PCa in PI-RADS 3	6	(60%)	5	(35.7%)	0.408
csPCa in PI-RADS 3	4	(40%)	3	(21.4%)	0.393
PCa in PI-RADS 4	95	(69.3%)	88	(67.7%)	0.793
csPCa in PI-RADS 4	61	(44.5%)	64	(49.2%)	0.463
PCa in PI-RADS 5	56	(88.9%)	44	(97.8%)	0.136
csPCa in PI-RADS 5	54	(85.7%)	40	(88.9%)	0.774

Abbreviation: CDR: cancer detection rate; csPCa: clinically significant prostate cancer; MRI: magnetic resonance imaging; PCa: prostate cancer; PI-RADS: Prostate Imaging Reporting and Data System; *p*: *p*-values representing the statistical significance of differences between internal and external MRIs.

**Table 4:** Comparative analysis of learning curves in MRI/US fusion-guided prostate biopsy outcomes: Urologist 1 vs. Urologist 2.

No. of UMFB	Urologist 1 (*n* = 157) *n*/*n* (%)				Urologist 2 (*n* = 242) *n*/*n* (%)				*p*
25	PI-RADS 3:	4	GS ≥ 7a:	0 (0%)	PI-RADS 3:	1	GS ≥ 7a:	0 (0%)	0.999
	PI-RADS 4:	13	GS ≥ 7a:	6 (46.1%)	PI-RADS 4:	16	GS ≥ 7a:	7 (43.8%)	0.999
	PI-RADS 5:	8	GS ≥ 7a:	7 (87.5%)	PI-RADS 5:	8	GS ≥ 7a:	4 (50%)	0.282
50	PI-RADS 3:	6	GS ≥ 7a:	2 (33.3%)	PI-RADS 3:	2	GS ≥ 7a:	0 (0%)	0.999
	PI-RADS 4:	25	GS ≥ 7a:	13 (52%)	PI-RADS 4:	33	GS ≥ 7a:	15 (45.5%)	0.791
	PI-RADS 5:	19	GS ≥ 7a:	17 (89.5%)	PI-RADS 5:	15	GS ≥ 7a:	10 (66.7%)	0.199
100	PI-RADS 3:	7	GS ≥ 7a:	2 (28.6%)	PI-RADS 3:	2	GS ≥ 7a:	0 (0%)	0.999
	PI-RADS 4:	64	GS ≥ 7a:	34 (53.1%)	PI-RADS 4:	75	GS ≥ 7a:	33 (44%)	0.310
	PI-RADS 5:	29	GS ≥ 7a:	26 (89.7%)	PI-RADS 5:	23	GS ≥ 7a:	18 (78.3%)	0.441
150	PI-RADS 3:	11	GS ≥ 7a:	5 (45.5%)	PI-RADS 3:	4	GS ≥ 7a:	0 (0%)	0.231
	PI-RADS 4:	99	GS ≥ 7a:	51 (51.5%)	PI-RADS 4:	104	GS ≥ 7a:	44 (42.3%)	0.207
	PI-RADS 5:	40	GS ≥ 7a:	35 (87.5%)	PI-RADS 5:	42	GS ≥ 7a:	35 (83.3%)	0.757
**Final count**	PI-RADS 3:	11	GS ≥ 7a:	5 (45.5%)	PI-RADS 3:	13	GS ≥ 7a:	2 (15.4%)	0.182
	PI-RADS 4:	104	GS ≥ 7a:	53 (51%)	PI-RADS 4:	163	GS ≥ 7a:	72 (44.2%)	0.315
	PI-RADS 5:	42	GS ≥ 7a:	37 (88.1%)	PI-RADS 5:	66	GS ≥ 7a:	57 (86.4%)	0.999

Final count = total number of MRI/US fusion-guided biopsies (UMFBs) performed by each urologist in this study (Urologist 1 = 157; Urologist 2 = 242); GS: Gleason score; No.: number; *p*: *p*-values representing the statistical significance of differences between Urologist 1 and Urologist 2 in achieving Gleason Score 7+ outcomes within each PI-RADS category at the specified biopsy counts; PI-RADS: Prostate Imaging Reporting and Data System; UMFB: prostate ultrasound/MRI fusion biopsy.

**Table 5:** Representation of significant deviations from Ahmed’s cutoffs per PI-RADS lesion for each urologist within the predefined case number ranges.

No. of UMFB	Urologist 1		Urologist 2	
	PI-RADS (Ahmed Cutoff)	*p*	PI-RADS (Ahmed Cutoff)	*p*
25	PI-RADS 3 (<20%)	0.999	PI-RADS 3 (<20%)	0.999
	PI-RADS 4 (>50%)	0.999	PI-RADS 4 (>50%)	0.804
	PI-RADS 5 (>80%)	0.999	PI-RADS 5 (>80%)	0.056
50	PI-RADS 3 (<20%)	0.345	PI-RADS 3 (<20%)	0.999
	PI-RADS 4 (>50%)	0.999	PI-RADS 4 (>50%)	0.728
	PI-RADS 5 (>80%)	0.400	PI-RADS 5 (>80%)	0.199
100	PI-RADS 3 (<20%)	0.633	PI-RADS 3 (<20%)	0.999
	PI-RADS 4 (>50%)	0.708	PI-RADS 4 (>50%)	0.356
	PI-RADS 5 (>80%)	0.249	PI-RADS 5 (>80%)	0.796
150	PI-RADS 3 (<20%)	0.050	PI-RADS 3 (<20%)	0.999
	PI-RADS 4 (>50%)	0.841	PI-RADS 4 (>50%)	0.141
	PI-RADS 5 (>80%)	0.322	PI-RADS 5 (>80%)	0.702
**Final count**	PI-RADS 3 (<20%)	0.050	PI-RADS 3 (<20%)	0.999
	PI-RADS 4 (>50%)	0.922	PI-RADS 4 (>50%)	0.158
	PI-RADS 5 (>80%)	0.247	PI-RADS 5 (>80%)	0.221

Final count = total number of MRI/US fusion-guided biopsies (UMFB) performed by each urologist in this study (Urologist 1 = 157; Urologist 2 = 242); No.: number; *p*: *p*-values representing the statistical significance of the violation of Ahmed’s criteria; PI-RADS: Prostate Imaging Reporting and Data System.

**Table 6:** Overview of studies evaluating learning curves in MRI/US fusion-guided biopsy.

First Author (Year)	Study Design, Methods, and Results
Yang et al. [25] (2024)	**Design**: single-center (UC), prospective (P), transperineal (TP); **Patients**: *n* = 92; **Urologist**: one experienced urologist, trained on a TP simulator prior to the study. **Statistical analysis**: CUSUM analysis for operating time with learning curve fitting, CDR, procedure duration, VAS, and VNS. **Endpoints**: CDR, procedure duration, and pain scores. **Results**: Two distinct phases identified: an initial learning phase (first 12 cases) followed by proficiency. No learning curve observed for csPCa CDR; statistically significant reduction in procedure duration and pain. **Level**: per urologist
Taha et al. [26] (2024)	**Design**: single-center (UC), retrospective (R), transrectal (TR); **Patients**: *n* = 403; **Urologists**: seven with varying experience levels (seniors ≥ 50 biopsies; juniors with no experience). **Statistical analysis**: linear regression and two-sample tests. **Endpoints**: CDR across prostate regions and junior vs senior urologist. **Results**: Consistent CDR across all experience levels; trend for positive correlation between experience and csPCa. No learning curve for csPCa CDR. **Level**: per urologist.
Ramacciotti et al. [27] (2024)	**Design**: single-center (UC), prospective (P), transperineal (TP); **Patients**: *n* = 370; **Urologist**: one highly experienced urologist. **Statistical analysis**: Wilcoxon rank sum, chi-square, logistic and linear regression, inflection point analysis. **Endpoints**: csPC CDR, procedure time, complication rates. **Results**: Learning curve for procedure time, plateau at 156 cases; consistently low complication rates. No learning curve for csPCa CDR. **Level**: per urologist.
Lenfant et al. [7] (2024)	**Design**: single-center (UC), prospective (P), transrectal (TR); **Patients**: *n* = 1721; **Urologists**: 14 with varying experience levels; novices supervised by lead operator. **Statistical analysis**: CUSUM. **Endpoints**: TRUS segmentation time, csPCa detection rate, pain score. **Results**: plateau for segmentation (40 cases), pain score (20–100 cases), and satisfactory csPCa CDR achieved after 25–48 cases. **Level**: per urologist.
Himmelsbach et al. [23] (2024)	**Design**: Single-center (UC), retrospective (R), transperineal (TP); **Patients**: *n* = 1716; **Urologists**: 20 with varying experience (low, intermediate, high). **Statistical analysis**: chi-square, Wilcoxon–Mann–Whitney U, regression analysis. **Endpoints**: CDR for csPCa in TB, procedure duration. **Results**: Significant decrease in procedure duration with experience. No learning curve for csPCa CDR. **Level**: institutional.
Alargkof et al. [28] (2024)	**Design**: single-center (UC), prospective (P), transperineal (TP); **Patients**: *n* = 91; **Urologists**: novice, chief resident, expert. **Statistical analysis**: Fisher’s exact test, t-test, logistic regression. **Endpoints**: CDR, procedure time, EPA scores, NASA task load index. **Results**: EPA scores plateau after 22 cases. No significant differences in CDR for PI-RADS 4 lesions for expert. No difference in CDR between surgeons **Level**: per urologist.
Xu et al. [8] (2023)	**Design**: Single-center (UC), retrospective (R), route unspecified; **Patients**: *n* = 107; **Urologists**: two board-certified urologists. **Statistical analysis**: CUSUM, t-test. **Endpoints**: CDR in PI-RADS 3, 4, and 5. **Results**: plateau for csPCa CDR observed after 52 biopsies; significant improvements in TB CDR. **Level**: institutional.
Hsieh et al. [15] (2023)	**Design**: single-center (UC), prospective (P), transperineal (TP); **Patients**: *n* = 206; **Urologist**: one experienced urologist. **Statistical analysis**: Cochrane–Armitage, McNemar test, logistic regression. **Endpoints**: temporal changes in csPCa CDR. **Results**: csPCa CDR improvements with time for TB; specific gains for lesions ≤ 1 cm and anterior lobe. **Level**: team-based.
Gereta et al. [5] (2023)	**Design**: single-center (UC), retrospective (R), transperineal (TP); **Patients**: *n* = 110; **Urologist**: one urologist inexperienced in UMFB. **Statistical analysis**: Kruskal–Wallis, chi-square, Fisher exact test, regression analysis. **Endpoints**: csPCa CDR, complication rates, biopsy core quality. **Results**: no learning curve for csPCa CDR; improvement in procedure time. **Level**: per urologist.
Calleris et al. [24] (2023)	**Design**: multi-center (MC), prospective (P), transperineal (TP); **Patients**: *n* = 1014; **Urologists**: 30 experienced (but various for perineal UMFB in LA). **Statistical analysis**: CUSUM, Kruskal–Wallis, chi-square, Jonckheere–Terpstra, linear regression. **Endpoints**: Biopsy duration, csCDR-T, complications, pain, urinary function. **Results**: plateau for procedure time at 50 biopsies; no significant trend for csCDR-T. **Level**: per urologist and institutional.
Görtz et al. [20] (2022)	**Design**: single-center (UC), prospective (P), transperineal (TP); **Patients**: *n* = 939; **Urologists**: 17 grouped by experience level. **Statistical analysis**: chi-square, Altman, Tango. **Endpoints**: RTB vs ETB csPCa CDR. **Results**: learning curve for csPCa CDR after 100 biopsies; better results in RTB vs ETB for low-experience group. **Level**: by experience level.
Urkmez et al. [19] (2021)	**Design**: single-center (UC), prospective (P), route unspecified; **Patients**: *n* = 1446; **Urologist**: one inexperienced urologist. **Statistical analysis**: Wilcoxon rank sum, Fisher’s exact, LOWESS. **Endpoints**: csCDR for Likert ≥ 3. **Results**: plateau reached at 500 cases; csPCa CDR significantly improved. **Level**: team-based.
Checcucci et al. [21] (2021)	**Design**: single-center (UC), prospective (P), transperineal (TP), and transrectal (TR); **Patients**: *n* = 1005; **Urologists**: two, inexperienced, but having undergone FGB training. **Statistical analysis**: ANOVA, linear and logistic regression. **Endpoints**: csPCa and PCa CDRs. **Results**: no influence of experience for csCDR, but stable CDRs for small lesions achieved after 100 procedures. **Level**: institutional
Cata et al. [18] (2021)	**Design**: single-center (UC), prospective (P), transrectal (TR); **Patients**: *n* = 400; **Urologists**: two with different levels of experience. **Statistical analysis**: chi-square, Kruskal–Wallis, multivariate regression. **Endpoints**: csPCa CDR for novice vs experienced. **Results**: improvement in csPCa CDR for experienced urologist only (after 52 cases). **Level**: per urologist.
Berg et al. [22] (2020)	**Design**: single-center (UC), prospective (P), transrectal (TR); **Patients**: *n* = 183; **Urologists**: experienced vs novice. **Statistical analysis**: logistic regression, IP weighting. **Endpoints**: CDR. **Results**: experience associated with higher odds of detecting PCa; no difference between expert and novice for CDR. **Level**: per urologist.
Kasabwala et al. [29] (2019)	**Design**: single-center (UC), retrospective (R), route unspecified; **Patients**: *n* = 173; **Urologist**: experienced in TR but not in UMFB. **Statistical analysis**: polynomial and linear regression, change point analysis. **Endpoints**: biopsy accuracy, specimen quality. **Results**: significant learning curve for biopsy accuracy up to 98 cases; no learning curve for csPCa CDR. **Level**: per urologist.
Truong et al. [30] (2018)	**Design**: Single-center (UC), retrospective (R), route unspecified; **Patients**: *n* = 113; **Urologists**: Five experienced in UMFB. **Statistical analysis**: Logistic regression, ROC. **Endpoints**: csCDR for PI-RADS 3–5. **Results**: Plateau in csPCa CDR for high PI-RADS lesions. **Level**: Institutional.
Meng et al. [16] (2018)	**Design**: single-center (UC), prospective (P), route unspecified; **Patients**: *n* = 1595; **Urologists**: four with varying experience. **Statistical analysis**: ANOVA, Kruskal–Wallis, trend test. **Endpoints**: csPCa CDR. **Results**: significant improvement in csPCa CDR over time. **Level**: institutional.
Mager et al. [14] (2017)	**Design**: single-center (UC), retrospective (R), transrectal (TR); **Patients**: *n* = 123; **Urologist**: one experienced, one novice. **Statistical analysis**: Wilcoxon–Mann–Whitney U, Kruskal-Wallis. **Endpoints**: detection quotient, procedure efficacy. **Results**: significant learning curve for TB detection quotient. **Level**: per urologist.
Calio et al. [17] (2017)	**Design**: single-center (UC), prospective (P), route unspecified; **Patients**: *n* = 1528; **Urologists**: cohort-based experience. **Statistical analysis**: McNemar, logistic regression. **Endpoints**: csPCa CDR. **Results**: increase in csPCa CDR, decrease in cisPCa. **Level**: cohort.
*****Own results*****	**Design**: single-center (UC), retrospective (R), access route not specified; **Patients**: *n* = 399; **Urologists**: two board-certified urologists experienced in prostate biopsies, yet new to UMFB. **Statistical analysis**: chi-square, Wilcoxon–Mann–Whitney U, binomial analysis. **Endpoints**: csCDR across PI-RADS categories 3, 4, and 5. **Results**: achieved optimal csPCa detection rates immediately following UMFB implementation. **Level**: per urologist.

Abbreviations: CDR: cancer detection rate; csCDR: CDR of clinically significant prostate cancer; CDR-T: cancer detection rate of clinically significant prostate cancer on target biopsy; cisPCa: clinically insignificant prostate cancer; csPCa: clinically significant prostate cancer; CUSUM: cumulative sum analysis; ETB: elastic image registration; FGB: fusion-guided biopsy; MC: multi-center; MRI: magnetic resonance imaging;; P: prospective; PCa: prostate cancer; PI-RADS: Prostate Imaging Reporting and Data System; R: retrospective; RTB: rigid image registration; TB: target biopsy; TP: transperineal; TR: transrectal; TRUS: transrectal ultrasound; UC: uni-center; US: ultrasound; VAS: visual analogue scale; VNS: visual numeric scale.

## Data Availability

The data sets generated and/or analyzed during the current study are available from the corresponding author on reasonable request.

## Abbreviations

C-CDR = cumulative cancer Detection rate; cisPCa = clinically insignificant prostate cancer; csPCa = clinically significant prostate cancer; DICOM = Digital Imaging and Communications in Medicine; DRE = digital rectal examination; ESUR = European Society of Urogenital Radiology; ISUP = International Society of Urological Pathology; mpMRI = multiparametric MRI; PACS = picture archiving and communication system; PCa = prostate cancer; PI-RADS = Prostate Imaging Reporting and Data System; PSA = prostate-specific antigen; RARP = robot-assisted radical prostatectomy; ROI = region of interest; SB = systematic biopsy; START = Standards of Reporting for MRI-targeted Biopsy Studies; TB = targeted biopsy; UMFB = ultrasound/MRI fusion biopsy of the prostate; U1 = urologist 1; U2 = urologist 2.

## References

[1] Cornford P., Bergh R.C.v.D., Briers E., Broeck T.V.D., Brunckhorst O., Darraugh J., Eberli D., De Meerleer G., De Santis M., Farolfi A. (2024). EAU-EANM-ESTRO-ESUR-ISUP-SIOG Guidelines on Prostate Cancer—2024 Update. Part I: Screening, Diagnosis, and Local Treatment with Curative Intent. Eur. Urol..

[2] Kasivisvanathan V., Rannikko A.S., Borghi M., Panebianco V., Mynderse L.A., Vaarala M.H., Briganti A., Budäus L., Hellawell G., Hindley R.G. (2018). MRI-Targeted or Standard Biopsy for Prostate-Cancer Diagnosis. N. Engl. J. Med..

[3] Klotz L., Chin J., Black P.C., Finelli A., Anidjar M., Bladou F., Mercado A., Levental M., Ghai S., Chang S.D. (2021). Comparison of Multiparametric Magnetic Resonance Imaging–Targeted Biopsy with Systematic Transrectal Ultrasonography Biopsy for Biopsy-Naive Men at Risk for Prostate Cancer: A Phase 3 Randomized Clinical Trial. JAMA Oncol..

[4] Ahmed H.U., El-Shater Bosaily A., Brown L.C., Gabe R., Kaplan R., Parmar M.K., Collaco-Moraes Y., Ward K., Hindley R.G., Freeman A. (2017). Diagnostic accuracy of multi-parametric MRI and TRUS biopsy in prostate cancer (PROMIS): A paired validating confirmatory study. Lancet.

[5] Gereta S., Hung M., Alexanderani M.K., Robinson B.D., Hu J.C. (2023). Evaluating the Learning Curve for In-office Freehand Cognitive Fusion Transperineal Prostate Biopsy. Urology.

[6] Halstuch D., Baniel J., Lifshitz D., Sela S., Ber Y., Margel D. (2019). Characterizing the learning curve of MRI-US fusion prostate biopsies. Prostate Cancer Prostatic Dis..

[7] Lenfant L., Beitone C., Troccaz J., Rouprêt M., Seisen T., Voros S., Mozer P.C. (2024). Learning curve for fusion magnetic resonance imaging targeted prostate biopsy and three-dimensional transrectal ultrasonography segmentation. BJU Int..

[8] Xu L., Ye N.Y., Lee A., Chopra J., Naslund M., Wong-You-Cheong J., Wnorowski A., Siddiqui M.M. (2022). Learning curve for magnetic resonance imaging/ultrasound fusion prostate biopsy in detecting prostate cancer using cumulative sum analysis. Curr. Urol..

[9] Barentsz J.O., Richenberg J., Clements R., Choyke P., Verma S., Villeirs G., Rouviere O., Logager V., Fütterer J.J. (2012). ESUR prostate MR guidelines 2012. Eur. Radiol..

[10] Padhani A.R., Weinreb J., Rosenkrantz A.B., Villeirs G., Turkbey B., Barentsz J. (2019). Prostate Imaging-Reporting and Data System Steering Committee: PI-RADS v2 Status Update and Future Directions. Eur. Urol..

[11] Sigle A., Jilg C.A., Kuru T.H., Binder N., Michaelis J., Grabbert M., Schultze-Seemann W., Miernik A., Gratzke C., Benndorf M. (2021). Evaluation of the Ginsburg Scheme: Where Is Significant Prostate Cancer Missed?. Cancers.

[12] Moore C.M., Kasivisvanathan V., Eggener S., Emberton M., Fütterer J.J., Gill I.S., Iii R.L.G., Hadaschik B., Klotz L., Margolis D.J. (2013). Standards of Reporting for MRI-targeted Biopsy Studies (START) of the Prostate: Recommendations from an International Working Group. Eur. Urol..

[13] Epstein J.I., Egevad L., Amin M.B., Delahunt B., Srigley J.R., Humphrey P.A. (2016). The 2014 International Society of Urological Pathology (ISUP) Consensus Conference on Gleason Grading of Prostatic Carcinoma: Definition of Grading Patterns and Proposal for a New Grading System. Am. J. Surg. Pathol..

[14] Mager R., Brandt M.P., Borgmann H., Gust K.M., Haferkamp A., Kurosch M. (2017). From novice to expert: Analyzing the learning curve for MRI-transrectal ultrasonography fusion-guided transrectal prostate biopsy. Int. Urol. Nephrol..

[15] Hsieh P.-F., Li P.-I., Lin W.-C., Chang H., Chang C.-H., Wu H.-C., Chang Y.-H., Wang Y.-D., Huang W.-C., Huang C.-P. (2023). Learning Curve of Transperineal MRI/US Fusion Prostate Biopsy: 4-Year Experience. Life.

[16] Meng X., Rosenkrantz A.B., Huang R., Deng F.-M., Wysock J.S., Bjurlin M.A., Huang W.C., Lepor H., Taneja S.S. (2018). The Institutional Learning Curve of Magnetic Resonance Imaging-Ultrasound Fusion Targeted Prostate Biopsy: Temporal Improvements in Cancer Detection in 4 Years. J. Urol..

[17] Calio B., Sidana A., Sugano D., Gaur S., Jain A., Maruf M., Xu S., Yan P., Kruecker J., Merino M. (2017). Changes in prostate cancer detection rate of MRI-TRUS fusion vs systematic biopsy over time: Evidence of a learning curve. Prostate Cancer Prostatic Dis..

[18] Cata E.D., Van Praet C., Andras I., Kadula P., Ognean R., Buzoianu M., Leucuta D., Caraiani C., Tamas-Szora A., Decaestecker K. (2021). Analyzing the learning curves of a novice and an experienced urologist for transrectal magnetic resonance imaging-ultrasound fusion prostate biopsy. Transl. Androl. Urol..

[19] Urkmez A., Ward J.F., Choi H., Troncoso P., Inguillo I., Gregg J.R., Altok M., Demirel H.C., Qiao W., Kang H.C. (2021). Temporal learning curve of a multidisciplinary team for magnetic resonance imaging/transrectal ultrasonography fusion prostate biopsy. BJU Int..

[20] Görtz M., Nyarangi-Dix J.N., Pursche L., Schütz V., Reimold P., Schwab C., Stenzinger A., Sültmann H., Duensing S., Schlemmer H.-P. (2022). Impact of Surgeon’s Experience in Rigid versus Elastic MRI/TRUS-Fusion Biopsy to Detect Significant Prostate Cancer Using Targeted and Systematic Cores. Cancers.

[21] Checcucci E., Piramide F., Amparore D., De Cillis S., Granato S., Sica M., Verri P., Volpi G., Piana A., Garrou D. (2021). Beyond the Learning Curve of Prostate MRI/TRUS Target Fusion Biopsy after More than 1000 Procedures. Urology.

[22] Berg S., Hanske J., von Landenberg N., Noldus J., Brock M. (2020). Institutional Adoption and Apprenticeship of Fusion Targeted Prostate Biopsy: Does Experience Affect the Cancer Detection Rate?. Urol. Int..

[23] Himmelsbach R., Hackländer A., Weishaar M., Morlock J., Schoeb D., Jilg C., Gratzke C., Grabbert M., Sigle A. (2024). Retrospective analysis of the learning curve in perineal robot-assisted prostate biopsy. Prostate.

[24] Calleris G., Marquis A., Zhuang J., Beltrami M., Zhao X., Kan Y., Oderda M., Huang H., Faletti R., Zhang Q. (2023). Impact of operator expertise on transperineal free-hand mpMRI-fusion-targeted biopsies under local anaesthesia for prostate cancer diagnosis: A multicenter prospective learning curve. World J. Urol..

[25] Yang Y., He X., Zeng Y., Lu Q., Li Y. (2024). The learning curve and experience of a novel multi-modal image fusion targeted transperineal prostate biopsy technique using electromagnetic needle tracking under local anesthesia. Front. Oncol..

[26] Taha F., Larre S., Branchu B., Kumble A., Saffarini M., Ramos-Pascual S., Surg R. (2024). Surgeon seniority and experience have no effect on CaP detection rates using MRI/TRUS fusion-guided targeted biopsies. Urol. Oncol. Semin. Orig. Investig..

[27] Ramacciotti L.S., Kaneko M., Strauss D., Hershenhouse J.S., Rodler S., Cai J., Liang G., Aron M., Duddalwar V., Cacciamani G.E. (2024). The learning curve for transperineal MRI/TRUS fusion prostate biopsy: A prospective evaluation of a stepwise approach. Urol. Oncol. Semin. Orig. Investig..

[28] Alargkof V., Engesser C., Breit H.C., Winkel D.J., Seifert H., Trotsenko P., Wetterauer C. (2024). The learning curve for robotic-assisted transperineal MRI/US fusion-guided prostate biopsy. Sci. Rep..

[29] Kasabwala K., Patel N., Cricco-Lizza E., Shimpi A.A., Weng S., Buchmann R.M., Motanagh S., Wu Y., Banerjee S., Khani F. (2019). The Learning Curve for Magnetic Resonance Imaging/Ultrasound Fusion-guided Prostate Biopsy. Eur. Urol. Oncol..

[30] Truong M., Weinberg E., Hollenberg G., Borch M., Park J.H., Gantz J., Feng C., Frye T., Ghazi A., Wu G. (2018). Institutional Learning Curve Associated with Implementation of a Magnetic Resonance/Transrectal Ultrasound Fusion Biopsy Program Using PI-RADS™ Version 2: Factors that Influence Success. Urol. Pr..

[31] Giganti F., Moore C.M. (2017). A critical comparison of techniques for MRI-targeted biopsy of the prostate. Transl. Androl. Urol..

[32] Stabile A., Dell’oglio P., Gandaglia G., Fossati N., Brembilla G., Cristel G., Dehò F., Scattoni V., Maga T., Losa A. (2018). Not All Multiparametric Magnetic Resonance Imaging–targeted Biopsies Are Equal: The Impact of the Type of Approach and Operator Expertise on the Detection of Clinically Significant Prostate Cancer. Eur. Urol. Oncol..

[33] Kornienko K., Reuter M., Maxeiner A., Günzel K., Kittner B., Reimann M., Hofbauer S.L., Wiemer L.E., Heckmann R., Asbach P. (2022). Follow-up of men with a PI-RADS 4/5 lesion after negative MRI/Ultrasound fusion biopsy. Sci. Rep..

[34] Arsov C., Becker N., Rabenalt R., Hiester A., Quentin M., Dietzel F., Antoch G., Gabbert H.E., Albers P., Schimmöller L. (2015). The use of targeted MR-guided prostate biopsy reduces the risk of Gleason upgrading on radical prostatectomy. J. Cancer Res. Clin. Oncol..

[35] Lee H., Hwang S.I., Lee H.J., Byun S.-S., Lee S.E., Hong S.K. (2018). Diagnostic performance of diffusion-weighted imaging for prostate cancer: Peripheral zone versus transition zone. PLoS ONE.

[36] Wegelin O., Exterkate L., van der Leest M., Kummer J.A., Vreuls W., de Bruin P.C., Bosch J., Barentsz J.O., Somford D.M., van Melick H.H. (2019). The FUTURE Trial: A Multicenter Randomised Controlled Trial on Target Biopsy Techniques Based on Magnetic Resonance Imaging in the Diagnosis of Prostate Cancer in Patients with Prior Negative Biopsies. Eur. Urol..

[37] Filson C.P., Natarajan S., Margolis D.J., Huang J., Lieu P., Dorey F.J., Reiter R.E., Marks L.S. (2016). Prostate cancer detection with magnetic resonance-ultrasound fusion biopsy: The role of systematic and targeted biopsies. Cancer.

[38] Drost F.-J.H., Osses D.F., Nieboer D., Steyerberg E.W., Bangma C.H., Roobol M.J., Schoots I.G. (2019). Prostate MRI, with or without MRI-targeted biopsy, and systematic biopsy for detecting prostate cancer. Cochrane Database Syst. Rev..

[39] Ahdoot M., Wilbur A.R., Reese S.E., Lebastchi A.H., Mehralivand S., Gomella P.T., Bloom J., Gurram S., Siddiqui M., Pinsky P. (2020). MRI-Targeted, Systematic, and Combined Biopsy for Prostate Cancer Diagnosis. N. Engl. J. Med..

[40] Rouvière O., Puech P., Renard-Penna R., Claudon M., Roy C., Mège-Lechevallier F., Decaussin-Petrucci M., Dubreuil-Chambardel M., Magaud L., Remontet L. (2019). Use of prostate systematic and targeted biopsy on the basis of multiparametric MRI in biopsy-naive patients (MRI-FIRST): A prospective, multicentre, paired diagnostic study. Lancet Oncol..

[41] Leitlinienprogramm Onkologie (2024). S3-Leitlinie Prostatakarzinom.

[42] Kaneko M., Medina L.G., Lenon M.S.L., Hemal S., Sayegh A.S., Jadvar D.S., Ramacciotti L.S., Paralkar D., Cacciamani G.E., Lebastchi A.H. (2023). Transperineal vs transrectal magnetic resonance and ultrasound image fusion prostate biopsy: A pair-matched comparison. Sci. Rep..

[43] Oderda M., Diamand R., Zahr R.A., Anract J., Assenmacher G., Delongchamps N.B., Bui A.P., Benamran D., Calleris G., Dariane C. (2024). Transrectal versus transperineal prostate fusion biopsy: A pair-matched analysis to evaluate accuracy and complications. World J. Urol..

[44] Mian B.M., Feustel P.J., Aziz A., Kaufman R.P., Bernstein A., Avulova S., Fisher H.A. (2024). Complications Following Transrectal and Transperineal Prostate Biopsy: Results of the ProBE-PC Randomized Clinical Trial. J. Urol..

